# META—Measurement for Evolution, Transformation, and Autorealization: A New Assessment Protocol

**DOI:** 10.3390/bs15070942

**Published:** 2025-07-11

**Authors:** Alessio Gori, Eleonora Topino

**Affiliations:** 1 Department of Health Sciences, University of Florence, Via di San Salvi 12, Pad. 26, 50135 Florence, Italy; 2 Integrated Psychodynamic Psychotherapy Institute (IPPI), Via Ricasoli 32, 50122 Florence, Italy; 3 Department of Human and Social Sciences, Mercatorum University, Piazza Mattei 10, 00186 Rome, Italy; eleonora.topino@gmail.com

**Keywords:** life satisfaction, psychological well-being, psychometrics, scale development, self-realization, self-actualization, self-fulfilment

## Abstract

Self-realization, a multifaceted concept, has long been a subject of interest in the scientific literature. Given its profound impact on overall well-being and work-related satisfaction, the development of instruments capturing its complexity assumes significant relevance. Therefore, this study presents the development and validation of the META—Measurement for Evolution, Transformation, and Autorealization, a self-report measure designed to assess the propensity for self-realization. The study involved a sample of 634 participants, who completed a survey comprising the META, Satisfaction with Life Scale, Career Adapt-Abilities Scale, General Self-Efficacy Scale, Insight Orientation Scale, and 10-item Connor–Davidson Resilience Scale. The analyses confirmed the statistical solidity of the three hypothesized sections; Part A (Evolutionary Thrust), including Sense of life, Spirit of service, Self-Authorizing, Self-Centering, and Internal Drive for Realization; Part B (Transformative Adaptation), including Propensity for transformation, Distress to change, Adaptability, and Fullness of the Experience; Part C (Work Attitude), including Social Service and Care, Administrative and Office Works, Entrepreneurship, Customer Service and Hospitality, and Manual activities. Factor analyses supported the structural validity of the three hypothesized sections of the META, and all subscales showed good to excellent internal consistency. Significant correlations between the META dimensions/subdimensions and self-realization or well-being outcomes also emerged. The META showed excellent psychometric properties and may be used in various fields, promoting advancements in research and practices supporting well-being and personal fulfilment.

## 1. Introduction

### 1.1. The Concept of Self-Realization

As early as Abraham Maslow’s Hierarchy of Needs, the concept of personal self-fulfilment was highlighted in its importance through the construct of self-actualization, which is located at the top of the pyramid and indicates the achievement of a person’s full potential and aspirations (Maslow, 1943, 1954). This model has been criticised, particularly regarding the rigid hierarchy of the needs pyramid; indeed, Maslow argued that self-actualization can only be attained after all lower-level needs are met, disregarding the fact that many people can pursue and achieve personal fulfilment even in the absence of satisfaction of all the lower-level needs (Wahba & Bridwell, 1976). Nonetheless, this theorization laid the foundation for further developments and contributed significantly to humanistic psychology, which today recognizes personal fulfilment as a key factor for well-being and personal satisfaction (Rogers, 1995; Deci & Ryan, 2000). In this regard, self-realization is a multifaced and complex construct that can be defined as the full development of individual potential, self-fulfilment, and authentic self-expression (Rogers, 1995). It is an ongoing process of personal growth and discovery (Deci & Ryan, 2000), associated with numerous positive outcomes well-documented in the scientific literature. Indeed, individuals with higher levels of self-realization tend to report greater well-being (Ryff, 2013), happiness (Seligman & Csikszentmihalyi, 2014; Schueller & Seligman, 2010), and satisfaction with life (Diener et al., 2018a, 2018b), to name a few.

### 1.2. Theoretical Background

#### 1.2.1. Self-Realization Within the Positive Psychology Framework

The empirical association between self-realization and indicators of well-being (Ryff, 2013) has led scholars to reconsider its centrality within the broader field of psychological health. In recent decades, this reconsideration has been especially shaped by the rise of positive psychology, a scientific movement that emerged in response to the traditional deficit-based approach of clinical psychology. Rather than focusing on dysfunction, positive psychology emphasizes the conditions that allow individuals and communities to thrive (Seligman & Csikszentmihalyi, 2000). Within this framework, self-realization is conceptualized not as a final achievement reserved for the few who have satisfied all basic needs, but as a fundamental psychological process that can unfold across the lifespan, even under challenging conditions (Linley et al., 2009).

This reconceptualization is closely linked to the notion of eudaimonia, which originates in Aristotelian philosophy and has been reintroduced into psychology as a counterpoint to hedonic models of well-being. Unlike hedonic well-being, which focuses on the pursuit of pleasure and the avoidance of discomfort, eudaimonic well-being is concerned with living in accordance with one’s true self, realizing one’s inner potential, and engaging in activities that are meaningful and socially valuable (Waterman et al., 2003; Ryan et al., 2008). Eudaimonia thus involves both authenticity and purpose, and presupposes an active engagement with one’s values and personal direction. In this view, self-realization is not merely a subjective state of satisfaction, but an ongoing and multidimensional process of growth and coherence that integrates affective, cognitive, and motivational components (Huta & Ryan, 2010; Schlegel et al., 2011).

In parallel, Self-Determination Theory (SDT) has provided a robust motivational framework for understanding how self-realization is supported and sustained. According to SDT, three innate psychological needs (autonomy, competence, and relatedness) constitute the foundation for optimal psychological functioning (Deci & Ryan, 2000; Ryan & Deci, 2017). When these needs are fulfilled, individuals are more likely to experience vitality, authenticity, and intrinsic motivation, which in turn foster engagement with personally meaningful goals. Self-realization, within this framework, can be seen as the process through which individuals internalize experiences and values, construct a coherent identity, and act in ways that reflect their true self (Sheldon, 2011). Crucially, this process is not static, but dynamic and adaptive, involving continuous re-evaluation and integration of life experiences in light of one’s evolving sense of meaning and purpose.

In this regard, meaning plays a pivotal role in self-realization. The search for meaning, and the capacity to find meaning in everyday life and in long-term goals, have been identified as central to eudaimonic well-being (Steger, 2012). Meaning provides structure and direction, particularly in the face of ambiguity, adversity, or change. The construction of meaning enables individuals to perceive coherence in their life narrative and to pursue goals that are not only personally relevant, but also socially significant (Wong, 2012; Martela & Steger, 2016). As such, the presence of meaning has been shown to play a crucial role in mediating the relationship between self-realization and various indicators of psychological adjustment and well-being (Steger, 2012).

Altogether, these contemporary contributions suggest that self-realization is best understood as a multidimensional construct that integrates various psychological processes. It encompasses an existential dimension, involving personal coherence, direction, and a sense of purpose; an adaptive dimension, reflecting the capacity to grow through complexity, uncertainty, and transformation; and a social-expressive dimension, where the realization of one’s self is enacted through vocational, relational, and civic engagement. This conceptualization aligns with findings from different strands of research that highlight the interaction between inner orientation and external action as essential to human development (Kegan, 1994; Joseph & Linley, 2006; Duffy et al., 2018).

Despite conceptual overlap, it is important to distinguish self-realization from related constructs such as psychological flourishing and self-transcendence. Flourishing refers to an overall state of optimal functioning that integrates emotional, psychological, and social well-being (Keyes, 2002), but does not necessarily imply an intentional pursuit of personal authenticity or growth. Self-transcendence, on the other hand, involves a movement beyond the boundaries of the self, often associated with spiritual, moral, or collective dimensions of meaning (Frankl, 1963; Wong, 2012). While both constructs intersect with aspects of self-realization, the latter maintains a distinct focus on the development of the self in alignment with personal values, aspirations, and context.

Given this complexity, there is a clear need for new tools capable of capturing the richness of self-realization in its modern, process-oriented understanding.

#### 1.2.2. Self-Realization in the Organizational Context

The self-realization process pervades all the dimensions of life, structuring identity (Hagmaier & Abele, 2012; Wrzesniewski et al., 1997), and giving meaning and purpose to life (Dik & Duffy, 2009). This implies that self-realization can potentially be pursued within any life role, including work (Duffy et al., 2018; Super, 1980; Schuurman, 2004). Employment can serve as a springboard for personal growth, development, and fulfilment (Steger et al., 2010), and achieving self-realization within one’s career is linked to various positive workplace outcomes. Previous research has shown that a high level of Self-Realization at work is correlated with greater job satisfaction (Judge et al., 2001). Furthermore, workers who perceive that they can realize their potential tend to be more committed, showing greater dedication and involvement (S. S. Kim et al., 2023; Schaufeli et al., 2006). Employees who feel fulfilled tend to show higher levels of creativity and innovation, contributing to the success and competitiveness of the organization (Carmeli et al., 2010). Furthermore, commitment resulting from a high level of self-realization can lead to greater productivity and an overall improvement in work performance (Luthans et al., 2015). Finally, a high level of self-realization is associated with lower turnover: when employees feel that their work contributes to their personal growth and the realization of their aspirations, they are less likely to leave the organization (Allen et al., 2010; Esteves & Lopes, 2017).

### 1.3. The Present Research

Given the significance of self-realization for overall and work-related well-being, various questionnaires have been developed for its measurement. Some of these instruments are one-dimensional, such as the Brief Calling Scale (Dik et al., 2012) and the Calling Scale (Dobrow & Tosti-Kharas, 2011). While these instruments offer valuable insights, their unidimensional structure may not adequately capture the complex and multifaceted nature of self-realization. Other measures adopt a multidimensional approach, such as the Calling and Vocation Questionnaire (Dik et al., 2012) and the Unified Multidimensional Calling Scale (Vianello et al., 2018), focusing on the motivational and affective dimensions of professional calling. Broader constructs, like self-actualization or eudaimonic well-being, have also been assessed through tools such as the Short Index of Self-Actualization (Jones & Crandall, 1986), Ryff’s Psychological Well-Being Scales (Ryff, 1989), and Purpose in Life questionnaires (Crumbaugh & Maholick, 1964). These instruments provide robust operationalizations of personal growth, autonomy, purpose, and meaning. However, they generally do not offer guidance on how self-realization may manifest across different domains of functioning, such as the workplace.

To the authors’ knowledge, there is currently a lack of instruments that both comprehensively assess the multidimensional psychological components of self-realization and identify the work-related orientations through which this potential may be expressed. This gap highlights the need for an integrative instrument that links the psychological processes of self-realization with vocational direction. Building on these insights, the general goal of the present study was to develop the *Measurement for Evolution, Transformation, and Autorealization (META)*, a new self-report instrument to assess subjective propensity to self-realization, making it applicable in career orientation, counselling, and therapy settings. The specific aims were as follows:

1. To develop the items of the META and assess its psychometric properties. This involved item generation, exploratory and confirmatory factor analysis, and internal consistency and reliability evaluation;
2. To examine the relationship between META scores and outcomes (work-related and non-work-related) typically associated with some self-realization. This included measures of life satisfaction, career adaptability, general self-efficacy, insight orientation, and resilience.

## 2. Materials and Methods

### 2.1. Participants, Procedure, and Ethics

A total sample of 634 subjects (73% female, 27% male) was involved in this research. They were aged from 18 to 86 years (*M_age_* = 37.32 years, *SD* = 14.97), and were recruited online through a snowball method, which was initiated via the researchers’ personal and professional networks. Participation was voluntary and devoid of any form of compensation. Each participant completed the Measurement for Evolution, Transformation, and Autorealization (META) and the other self-report measures along with a demographic questionnaire (sex, age, marital status, occupation, and degree of study) online on the Google Forms platform, after they were informed about the general aim of the research and provided informed consent electronically. Privacy and anonymity were guaranteed. The study was approved by the research team’s institutional Ethical Committee.

### 2.2. Development of the Measurement for Evolution, Transformation, and Autorealization (META)

The Measurement for Evolution, Transformation, and Autorealization (META) was conceptualized with the following intent:

1. Being jargon-free and avoiding colloquialisms, to adapt to different cultural contexts and educational levels;
2. Being of easy use (i.e., being agile both in the administration and in the scoring);
3. Being useful in different phases of personal and work orientation and maintaining an interdisciplinary scope (work orientation, psychological treatment, psychotherapy, etc.).

With this in mind, the first step in scale development was the definition of the constructs for measurement and the generation of the pool of candidate items, according to international guidelines (Netemeyer et al., 2003). In light of this, an exploration and integration of the scientific literature relating to the propensity towards self-realization was carried out, on the basis of which the structure of the Measurement for Evolution, Transformation, and Autorealization (META) was defined as consisting of three sections: Part A (*Evolutionary Thrust*), Part B (*Transformative Adaptation*), and Part C (*Work Attitude*).

- *Evolutionary Thrust* (Part A) concerns the intrinsic evolutionary drive that guides individuals or systems towards realizing their potential and fostering progress in themselves and others (Banathy, 1987). The focus of this part was on the abstract, internal, and profound dynamics that can influence the propensity for self-realization. Specifically, five subdimensions were included:
  - Sense of life, i.e., the perception and awareness of one’s life’s purpose, meaning, and direction. This concept encompasses the subjective experience of one’s life as coherent and significant, and is often associated with notions of fulfilment and personal significance (Wong, 2015; Steger et al., 2021). Research consistently demonstrates a strong connection between a sense of life and enhanced well-being. Indeed, having a strong sense of life is positively correlated with happiness and negatively related to anxiety and depression (Crego et al., 2021). Additionally, previous evidence suggests that purpose in life may lead to better physical health outcomes, such as reduced risk of chronic diseases and improved longevity (Martela et al., 2024; Musich et al., 2018).
  - Spirit of service, i.e., a deeply ingrained motivation to support others, which is often driven by altruistic values. This concept involves the desire to dedicate oneself to the well-being of others (Loizzo et al., 2012). Altruism and helping behaviors can also be a source of personal growth and well-being (Seligman & Csikszentmihalyi, 2000). Indeed, previous research has shown significant and positive correlations between prosociality and emotional, relational, and life satisfaction (Buunk & Schaufeli, 1999; C. Lu et al., 2021; Weinstein & Ryan, 2010).
  - Self-Authorizing, i.e., the sense of personal authorization in making independent decisions and choices, granting themselves the authority to determine their actions and paths in life. This concept is critical in fostering a sense of control and agency (Oshana, 2014). Autonomy, a core component of self-authorizing, supports the development of self-respect and personal fulfilment, which are critical for emotional and psychological health (Johnston, 2021). Consistently, previous research showed that fostering a sense of self-authority and personal autonomy may contribute to emotional stability and life satisfaction (O’Hara & Lyon, 2014).
  - Self-Centering, i.e., a state of alignment where individuals are deeply connected with their passions, interests, and personal identity through their activities. This connection creates a state of harmony and resonance between an individual’s inner self and their external actions (Franklin, 2022). Previous research has explored the importance of this factor in the workplace. For example, existing evidence shows that the alignment between personal and professional identity is associated with greater job satisfaction (Zhang et al., 2018), lower risk of burnout (Rasmussen et al., 2018), and better job outcomes (Fitzgerald, 2020).
  - Internal drive for realization, i.e., the intrinsic motivation to achieve personal fulfilment, seek a life purpose, and evolve. This drive supports personal development and growth, as it propels individuals towards continuous self-improvement and the attainment of meaningful goals (Weinstein et al., 2013). The internal drive for realization encourages individuals to continuously develop their skills, knowledge, and abilities (Bedan et al., 2021; Palamarchuk & Haba, 2023). This process of personal development is essential for achieving long-term goals and improving overall well-being (Maksimenko & Serdiuk, 2016; Waterman et al., 2003)
- *Transformative Adaptation* (Part B) concerns the ability to create new pathways to facilitate development, growth, and evolution (see Ajulo et al., 2020 for a review). The focus of this part was on experiential, external and behavioral dynamics, which are practically useful for self-realization. Specifically, four subdimensions were included:
  - Propensity for transformation, i.e., an inclination to actively seek, welcome, and engage in significant life changes aimed at personal growth and development. Individuals with a high propensity for transformation are often characterized by their proactive approach to life changes: they are not only open to new experiences but actively seek them out, driven by a desire for self-improvement (Di Fabio & Gori, 2016a). Research consistently shows that individuals with a high propensity for transformation are more likely to turn adversity into opportunities for personal development, exhibiting greater posttraumatic growth (Lepore & Revenson, 2014). Consistently, longitudinal studies have highlighted that openness to experience, which correlates with a propensity for transformation, can lead to significant personality development and positive changes over time (Caspi & Roberts, 2001).
  - Distress to change (the only reverse subdimension), i.e., the tendency to resist or struggle with changes in life, a general reluctance to embrace change. This aversion can manifest in various ways, including emotional discomfort, behavioral rigidity, and cognitive inflexibility (Ridner, 2004). Individuals with high levels of distress in response to changes often experience increased levels of burnout and emotional illness (Sablonnière et al., 2012). The inability to adapt to new situations can lead to a sense of helplessness and decreased psychological well-being (Oreg, 2003).
  - Adaptability, i.e., the ability to adjust to new conditions, alter one’s path when necessary, and change habits in response to changing circumstances. This construct reflects a person’s flexibility, problem-solving skills, and resilience (Coşkun et al., 2014; Nakhostin-Khayyat et al., 2024). The ability to adjust to new and challenging situations can help to effectively manage emotional distress (Bocciardi et al., 2017), fostering hope, optimism, and life satisfaction (Santilli et al., 2017; Liu et al., 2013). Furthermore, it can enhance tolerance of uncertainty, directing efforts to understand uncertain situations into creative thoughts and actions (Beghetto, 2019; Orkibi, 2021).
  - Fullness of the experience, i.e., the propensity to capture the richness and depth of an individual’s engagement with life. This concept highlights a motivation to learn and gain insights from lived experiences (Csikszentmihalhi, 2020). This mindset may lead to ongoing self-improvement and the acquisition of new skills and perspectives, favoring personal growth (Hood & Carruthers, 2007). Indeed, by engaging deeply with their experiences, individuals cultivate new skills and broaden their perspectives. This approach not only enhances their problem-solving abilities but also enriches their overall life satisfaction and well-being (Csikszentmihalhi, 2020).
- *Work Attitude* (Part C) is related to work orientation with respect to different types of activities and sectors. Specifically, five subdimensions were included:
  - Social Service and Care, i.e., jobs in helping professions and social settings. Examples include working in nursing homes, providing support services, social work, nursing, working with individuals in need of assistance, community work, teaching, and childcare.
  - Administrative and Office Works, i.e., occupations in which tasks commonly associated with office environments are performed. Examples include administration, accounting, computer proficiency, working with numbers, and experience in banking, public services, technology, and advertising.
  - Entrepreneurship, i.e., self-directed work and professions that involve starting or managing businesses. Examples include freelancers, engineers, architects, lawyers, doctors, psychologists, sports professionals, traders, and retail business owners (clothing/jewelry shops).
  - Customer Service and Hospitality, i.e., jobs requiring effective interaction with customers in tourism, including service-oriented roles. Examples include work in hotels, restaurants, bars, nightclubs, bed and breakfasts, souvenir shops, street vending, and tour guides.
  - Manual activities, i.e., occupations in which hands-on tasks, including those involved in construction, manufacturing, transportation, and maintenance, are performed. Examples include laborers, industrial workers, drivers, farmers, painters, sculptors, carpenters, bricklayers, cleaning service personnel, electricians, and plumbers.

The propensity for each of these work areas is investigated through questions assessing the following: (1) Pleasure in the Activity, referring to enjoyment of work tasks, which has been significantly associated with enhanced productivity and career satisfaction (Graves et al., 2012; Frederick & Lazzara, 2020); (2) Perception of Being Able to Perform the Activity, referring to confidence and the sense of professional self-efficacy, which has been significantly associated with higher engagement in challenging job demands, quality in decision-making processes, and job satisfaction (M. Lu et al., 2020; Gori et al., 2022b; Ventura et al., 2015); (3) Desire to Perform the Activity, referring to the genuine interest and intrinsic desire to engage in specific professional activities, which has been significantly associated with organizational commitment and job performance (Joo et al., 2010; S. S. Kim et al., 2023); (4) Commitment to the Activity, referring to the dedication and responsibility employees feel towards their job roles, which has been significantly associated with lower turnover intentions and higher organizational effectiveness (Guzeller & Celiker, 2020; Yadav et al., 2022).

The item generation process was conducted through multiple focus groups involving a panel of six experts in psychological assessment, psychotherapy, and work and organizational psychology. The aim was to ensure strong theoretical alignment with the constructs underlying the scale. Each subdimension was operationalized into a set of items, which were developed through collaborative discussion and iterative refinement during these sessions. The development of this scale was guided by three key criteria: (1) Conceptual grounding, i.e., subscales within each section were meticulously conceptualized based on established theoretical foundations from the relevant scientific literature; (2) Clarity and Conciseness, i.e., items were crafted to be brief, specific, clear, and straightforward for optimal comprehension; (3) Administrative Efficiency, i.e., the number of items was carefully chosen to maximize the speed and ease of scale administration: three items for each subdimension in Part A (Evolutionary Thrust) and Part B (Transformative Adaptation), and four items for each work area in Part C (Work Attitude). All items generated during the focus groups followed a process of collaborative evaluation and revision. Specifically, each item was discussed and refined until full consensus was reached among the experts regarding its conceptual relevance, clarity, and capacity to represent the intended dimension. This approach ensured that each item contributed meaningfully to the measurement of the target construct without redundancy or ambiguity. The response format for all items was a 5-point Likert scale (1 = “not at all”, 2 = “only a little”, 3 = “to some extent”, 4 = “rather much”, 5 = “very much”), chosen for its clarity, ease of interpretation, and compatibility with the psychometric aims of the scale. For each subdimension, scores were calculated by summing the corresponding items. In Parts A and B, total scores were also computed to capture the broader latent dimensions, in line with reflective measurement models and the high internal consistency observed.

### 2.3. Measures

#### 2.3.1. Measurement for Evolution, Transformation, and Autorealization (META)

The *Measurement for Evolution, Transformation, and Autorealization (META)* is a battery of self-report scales used to assess the subjective propensity to self-realization. It consists of three sections: (1) Part A (Evolutionary Thrust; 15 items) explores the internal dynamics and profound experiences that can be the basis of the search for one’s self-realization, and allows for the calculation of both subscale scores (i.e., Sense of life, Spirit of service, Self-Authorizing, Self-Centering, Internal drive for realization) and a total score; Part B (Transformative Adaptation; 12 items) evaluates the behavioral and external dynamics that may be practically relevant for self-realization, and allows for the calculation of both subscale scores (i.e., Propensity for transformation, Distress to change, Adaptability, Fullness of the experience) and a total score; Part C (Work Attitude; 20 items) evaluates the propensity towards specific job areas, and allows for the calculation of scores concerning the 5 sectors explored (i.e., Social Service and Care, Administrative and Office Works, Entrepreneurship, Customer Service and Hospitality, Manual activities), but there is no total score. All the items are rated on a 5-point Likert scale, from 1 (“*not at all*”) to 5 (“*very much*”). In the present sample, both the total scores (for parts A and B) and the subscales showed good internal consistency (see the Cronbach’s alpha and McDonald’s omega coefficients reported in the Results section).

#### 2.3.2. Satisfaction with Life Scale (SWLS)

The *Satisfaction with Life Scale* (SWLS; Diener et al., 1985; Italian version: Di Fabio & Gori, 2016b, 2020) is a 5-item self-report measure designed to evaluate global life satisfaction. Items are rated on a 7-point Likert scale ranging from 1 (“*Strongly disagree”*) to 7 (“*Strongly agree*”). The Italian version used in this study showed good internal consistency (*α* = 0.878, *ω* = 0.890).

#### 2.3.3. Career Adapt-Abilities Scale (CAAS)

The *Career Adapt-Abilities Scale* (CAAS; Savickas & Porfeli, 2012; Italian version: Soresi et al., 2012) is a 24-item self-report measure designed to evaluate career adaptability and its components. Items are scored on a 5-point Likert scale, ranging from 1 (“*Not a strength*”) to 5 (“*Greatest strength*”). The scale allows for both a total score (higher values indicating higher levels of career adaptability) and the scores concerning its four subdimensions (Concern, Control, Curiosity, and Confidence). The Italian version used in this study showed good internal consistency for both the total (*α* = 0.939, *ω* = 0.942) and the subscale scores (*α* = 0.894 and *ω* = 0.896 for Concern, *α* = 0.810 and *ω* = 0.818 for Control, *α* = 0.863 and *ω* = 0.864 for Curiosity, *α* = 0.896 and *ω* = 0.898 for Confidence).

#### 2.3.4. General Self-Efficacy Scale (GSES)

The *General Self-Efficacy Scale* (GSES; Schwarzer & Jerusalem, 1995; Italian version: Sibilia et al., 1995) is a 10-item self-report measure designed to evaluate self-efficacy. Items are rated on a 4-point Likert scale, ranging from 1 (“*not at all true for me*”) to 4 (“*very true for me*”). The Italian version used in this study showed good internal consistency (*α* = 0.818, *ω* = 0.884).

#### 2.3.5. Insight Orientation Scale (IOS)

The *Insight Orientation Scale* (IOS; originally developed in Italian: Gori et al., 2015, 2022d) is a 7-item self-report measure designed to evaluate the individual orientation towards insight, considering its constitutive components and manifestations. Items are scored on a 5-point Likert scale, ranging from 1 (“*not at all*”) to 5 (“*a great deal*”). The Italian version used in this study showed good internal consistency (*α* = 0.783, *ω* = 0.790).

#### 2.3.6. 10-Item Connor–Davidson Resilience Scale (I-CD-RISC-10)

The *10-item Connor–Davidson Resilience Scale* (I-CD-RISC-10; Campbell-Sills & Stein, 2007; Italian version: Di Fabio & Palazzeschi, 2012) is a 10-item self-report measure designed to assess the degree of the respondent’s resilience. Items are scored on a 6-point Likert scale, ranging from 0 (“*Not true at all*”) to 5 (“*True nearly all the time*”). The Italian version used in this study showed good internal consistency (*α* = 0.848, *ω* = 0.855).

### 2.4. Data Analysis

Statistical analyses were conducted using Jamovi 2.2.21 (The jamovi project, 2024; Sydney, Australia), SPSS (v. 21.0; IBM, New York, NY, USA), and AMOS (v. 24.0; IBM, New York, NY, USA). Descriptive statistics were calculated. Item analysis for part A, B, and C of the META was performed to check the normality of data distribution: for sample sizes greater than 300, an absolute skew value equal to or less than 2 or an absolute kurtosis value equal to or less than 7 may be interpreted as indicative of normality (H. Y. Kim, 2013). The Kaiser–Meyer–Olkin (KMO) statistic and Bartlett’s test of sphericity were used to assess sampling adequacy for factor analysis, supporting the data suitability when the KMO value is more than 0.7 and Bartlett’s test is statistically significant (*p* < 0.001; Mulaik, 2009). To investigate the dimensionality of the META, the sample was split into two subsamples (*n* = 317) using simple random sampling procedures. In the first, Exploratory Factor Analyses (EFAs) with a “Principal axis factoring” extraction method were used in combination with a “Promax” rotation, identifying the number of factors for each META section based on parallel analysis. In the second subsample, Confirmatory Factor Analyses (CFAs) were performed to assess the fit of the measurement models to the data, considering the following indices: the chi-square (*χ*^2^) of the model, indicating a good fit when the probability value is statistically non-significant (*p* < 0.05; Hooper et al., 2008); the discrepancy divided by degree of freedom (CMIN/DF), indicating a reasonable fit for values less than 5 (Marsh & Hocevar, 1985); the Goodness of Fit (GFI), Tucker Lewis index (TLI), and the Comparative Fit Index (CFI), indicating a reasonable fit for values above 0.90 (Hu & Bentler, 1999; Kline, 2015); the Root Mean Square Error of Approximation (RMSEA) and Standardized Root Mean Square Residual (SRMR), indicating reasonable fit for values values less than 0.08, and poor fit for values greater than 0.10 (Fabrigar et al., 1999). Furthermore, the factorial structure of each section was further tested through a comparison with unifactorial solutions by using the Δ*χ*^2^, with significant values indicating a statistically significant difference in the fit of the models to the data (Byrne, 2010). To assess the internal consistency of each META section, the Cronbach’s alpha (*α*; Cronbach, 1951) and McDonald’s omega (*ω*; McDonald, 2013) coefficients were calculated. Pearson’s correlation was used to explore convergent and discriminant validity. The latter was also investigated through the HTMT analysis, performed through an AMOS plugin (Gaskin & James, 2019), indicating a satisfactory distinguishability of the constructs evaluated by the META scales when the values were below 0.85 (Henseler et al., 2015).

## 3. Results

### 3.1. Preliminary Analyses

Descriptive statistics of the sample are shown in Table 1.

Additional descriptive statistics for each item, including mean, standard deviation, percentage endorsement by response category, and item-total correlations, are reported in Supplementary Tables S1–S3.

The item analysis suggested an approximately normal distribution, with skewness values between −1 and +1, and absolute kurtosis values ranging from 0.090 to 4.729 (below the cut-off of 7).

KMO values of 0.823 (Part A), 0.765 (Part B), and 0.790 (Past C), as well as the statistically significant values of Bartlett’s test for each META section, supported the data’s suitability for factor analysis.

### 3.2. Factor Analysis and Internal Consistency

**Figure 1:**
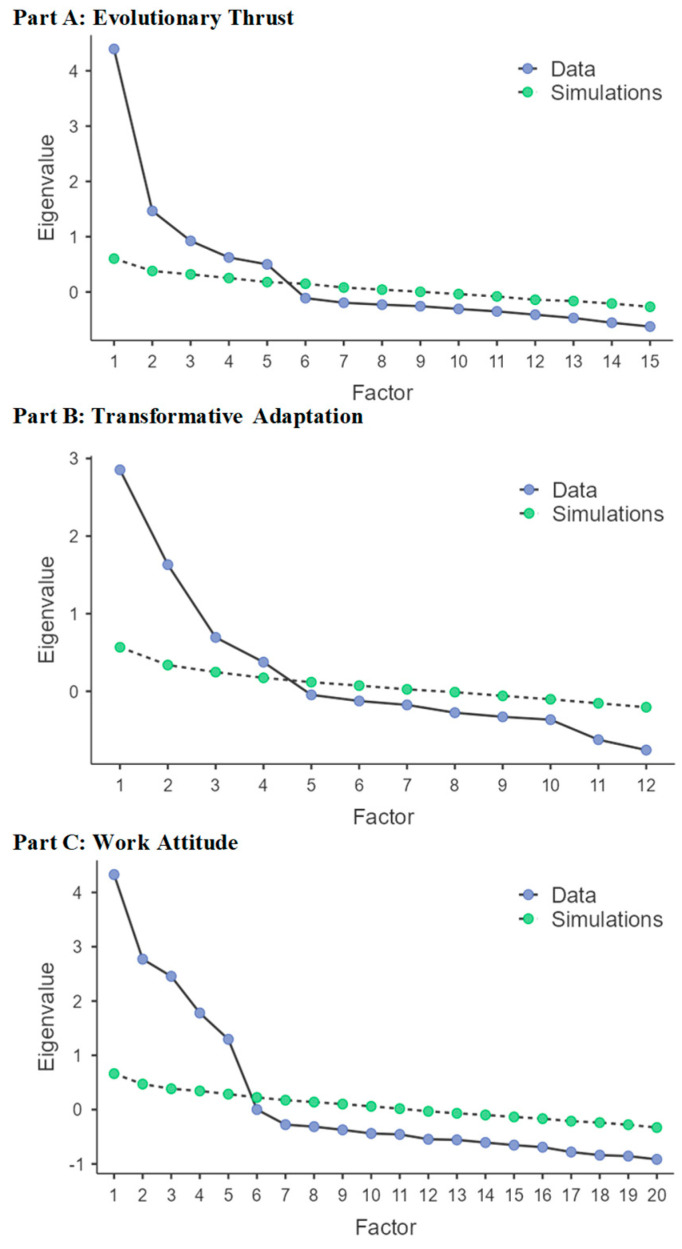
Scree plot of parallel analysis for each section of the META, using eigenvalues from META data (blue slope) and random data (green slope).

Exploratory factor analyses (EFAs) for each META section yielded five (Part A), four (Part B), and five (Part C) interpretable factors, which explained 65.6%, 58.2%, and 77.2% of the total variance, respectively (see Table 2 and Figure 1).

**Figure 2:**
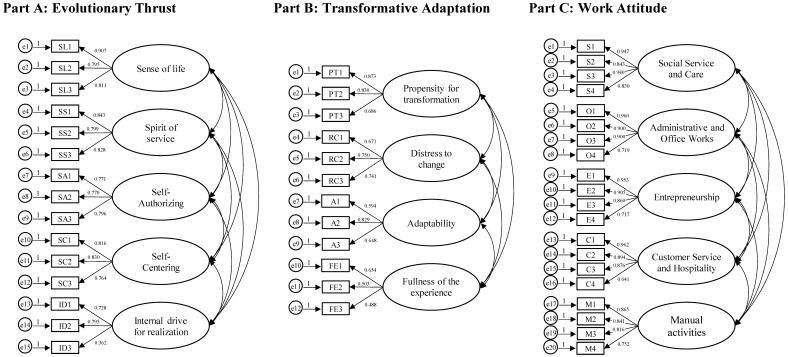
CFAs results for Part A, Part B, and Part C of the META.

The Confirmatory factor analyses (CFAs, see Figure 2) showed that the obtained factorial solutions provided acceptable fit to the data for each META section. Specifically, for Part A, the Chi-square was statistically significant (*p* < 0.001), but the other indices showed acceptable values: CMIN/DF = 2.124, GFI = 0.935, TLI = 0.945, CFI = 0.958, RMSEA = 0.060, and SRMR = 0.065. Similarly, the indices of part B were *χ*^2^ = 165.171 (*p* < 0.001) CMIN/DF = 3.441, GFI = 0.927, TLI = 0.863, CFI = 0.900, RMSEA = 0.088, and SRMR = 0.069. Finally, the indices of Part C were *χ*^2^ = 561.338 (*p* < 0.001) CMIN/DF = 3.508, GFI = 0.841, TLI = 0.912, CFI = 0.926, RMSEA = 0.089, and SRMR = 0.044. Furthermore, the adequacy of the obtained factorial structure for each section was confirmed by testing its fit superiority compared with the unifactorial models through the exploration of the chi-square difference test, which was found to be significant and confirmed the goodness of the correlational models (see Table 3).

Concerning the internal consistency of the META, Cronbach’s alpha and McDonald’s omega coefficients showed satisfactory values for all three sections (see Table 2): Part A (*α* = 0.806 and *ω* = 0.828 for the total score), Part B (*α* = 0.721 and *ω* = 0.754 for the total score), and Part C (values ranging from *α* = 0.945, *ω* = 0.945 to *α* = 0.887, *ω* = 0.888).

### 3.3. Convergent and Discriminant Validity

With regard to convergent validity, Pearson’s r analysis showed significant and positive associations between the Total score of the META Part A and Satisfaction with life (*r* = 0.526, *p* < 0.01), Career adaptability (*r* = 0.613, *p* < 0.01), General self-efficacy (*r* = 0.445, *p* < 0.01), Insight orientation (*r* = 0.531, *p* < 0.01), and Resilience (*r* = 0.438, *p* < 0.01). Total score of the META Part B was significantly and positively correlated with Career adaptability (*r* = 0.353, *p* < 0.01), General self-efficacy (*r* = 0.380, *p* < 0.01), Insight orientation (*r* = 0.341, *p* < 0.01), and Resilience (*r* = 0.442, *p* < 0.01).

Concerning Part C, the first factor (Social Service and Care) was significantly and positively associated with Insight orientation (*r* = 0.085, *p* < 0.05), and Resilience (*r* = 0.103, *p* < 0.01); the second factor (Administrative and Office Works) showed a significant and positive association with Career adaptability (*r* = 0.090, *p* < 0.05); the third factor (Entrepreneurship) was significantly and positively correlated with Career adaptability (*r* = 0.193, *p* < 0.01), General self-efficacy (*r* = 0.247, *p* < 0.01), Insight orientation (*r* = 0.222, *p* < 0.01), and Resilience (*r* = 0.212, *p* < 0.01); the fourth factor (Customer Service and Hospitality) was significantly and positively associated with Resilience (*r* = 0.100, *p* < 0.05); the fifth factor (Manual Activities) did not show significant correlations with other scale totals, but was significantly and positively associated with a subscale of the Career Adapt Ability Scale, i.e., Control (*r* = 0.086, *p* < 0.05).

The HTMT inference did not indicate problems of discriminant validity for the META: its associations with the other scales (see Table 4) and those between the factors of each part (see Table 2) are below the threshold value of 0.85.

## 4. Discussion

Multiple lines of research have identified self-realization as a highly relevant factor, emphasizing its significance for both work outcomes and subjective well-being (see Duffy et al., 2018; Duffy & Dik, 2013; Martela & Pessi, 2018 for reviews). Accordingly, developing assessment tools that are versatile across various life contexts and which may capture the complexity of this construct represents a key base for research and counselling practices on this issue (e.g., Duffy et al., 2015). Based on this rationale, the present research aimed at developing the *Measurement for Evolution, Transformation, and Autorealization (META),* a new self-report instrument to assess the subjective propensity to self-realization, and explore its psychometric properties.

### 4.1. META: Development and Exploration of the Psychometric Properties

The META demonstrated excellent psychometric properties, including strong evidence for both validity and reliability. The process of conceptualization and development of the META was implemented in light of the scientific evidence on the topic of self-realization, resulting in a self-report questionnaire divided into three sections: *Evolutionary Thrust* (Part A, 15 items, 5 subscales) focuses on the intrinsic drive for growth and progress, both personally and for others (Banathy, 1987); *Transformative Adaptation* (Part B, 12 items, 4 subscales) explores an individual’s tendency to create new approaches and strategies to facilitate development and growth (see Ajulo et al., 2020 for a review); *Work Attitude* (Part C, 20 items, 5 subscales), which delves into work orientations across various activities and sectors.

The subdivision into three parts, each comprising four or five factors, is supported by factor analyses (both EFA and CFA), and is in line with the multidimensionality of the concept as described by the scientific literature (e.g., Duffy et al., 2018; Hagmaier & Abele, 2012; Praskova et al., 2015). This categorization therefore reflects the complexity of the construct of the propensity for self-realization, which finds application in different fields of the individual’s life, including work (Duffy et al., 2023; Hirschi et al., 2018). Furthermore, both the total scores for each section and the respective subscales exhibit strong internal consistency (Cronbach, 1951; McDonald, 2013), indicating a high degree of cohesion within each dimension. In parallel, the questionnaire showed good evidence of discriminant validity (Henseler et al., 2015), providing support for the robust psychometric properties of this instrument and its reliability in evaluating distinct aspects of self-realization propensity.

This was further supported by the associations between the META and the measures used to assess concurrent validity, i.e., life satisfaction, career adaptability, general self-efficacy, insight orientation, and resilience.

### 4.2. Association Between the META and Self-Realization or Well-Being Outcomes

Consistently with previous research (e.g., Wolfram, 2023), satisfaction with life was significantly and positively correlated with Evolutionary Thrust, both considering its total score and in most of its subdimensions. The only factor within Part A showing a negative association was the Internal Drive for Realization. This may suggest that the search for fulfilment and meaning may be fuelled by a state of dissatisfaction with the current state (Park et al., 2010). From a dialectical perspective, this tension may not indicate lower well-being per se, but rather reflect an active engagement with personal growth. In fact, psychological development often involves moments of discomfort, ambiguity, or internal conflict that can serve as catalysts for transformation and deeper self-exploration (Carver, 1998; Wong, 2011). Therefore, individuals with a stronger internal drive may experience a motivational imbalance between their current state and their ideal self, which could temporarily reduce life satisfaction while supporting long-term self-realization processes. The same reading can be given to the negative correlation between Satisfaction with life and Propensity for Transformation (part B). Within this section of the META, Distress to Change was also significantly and negatively correlated to life satisfaction, and this confirms and expands upon previous evidence showing the positive relationship between acceptance of change and well-being (Di Fabio et al., 2016). On the other hand, both adaptability and fullness of the experience showed significant and positive connections with satisfaction with life, echoing existing evidence (Csikszentmihalhi, 2020; Shi et al., 2015). Remarkably, none of the Work Attitudes (Part C) were associated with satisfaction with life, which therefore were not linked to a specific work area per se, but could instead be connected to the realization of one’s personal aspirations (Bialowolski & Weziak-Bialowolska, 2021). This finding should be interpreted in light of the fact that Part C does not assess whether individuals are actually engaged in the corresponding work domains, but rather their inclination or perceived affinity toward those areas. As such, a misalignment between one’s vocational preferences and real-life occupational context may weaken associations with well-being indicators. Future studies should explore whether congruence between vocational orientation and actual job role moderates the relationship between work attitudes and life satisfaction.

Regarding career adaptability, this dimension exhibited significant positive correlations with both Part A (Evolutionary Thrust) and Part B (Propensity for Transformation), aligning with the scientific literature (Topino et al., 2022). These findings suggest that a stronger sense of self-fulfilment facilitates the successful management of one’s professional development (Savickas, 1997) by enabling individuals to effectively navigate the pressures and conditions encountered in various work environments (Super & Knasel, 1981). Career Adaptability was only negatively associated with Distress to Change, which can in fact be a source of greater stress and dissatisfaction in the work context (Paloş et al., 2022; Turgut et al., 2016). Furthermore, Career Adaptability correlated with the orientation towards administrative and office works and entrepreneurship. Indeed, individuals with high career adaptability are capable of quickly adapting to changes in work dynamics, roles, and responsibilities within an office environment (Kaur & Kaur, 2020). On the other hand, in entrepreneurship, adaptability is essential to navigate the uncertainties and constant changes associated with market trends, customer needs, and technological advancements (Atitsogbe et al., 2019).

Concerning Self-Efficacy, this dimension was positively correlated with both Part A (Evolutionary Thrust) and Part B (Propensity for Transformation), except for Internal Drive for Realization, Propensity for Transformation, and Distress to Change. These results are consistent with previous research results about the relationship between self-efficacy, which showed positive associations with Presence of Meaning, and a negative relationship with Search for Meaning (Gori et al., 2022c). Similar to the associations observed with life satisfaction, a state of personal dissatisfaction can potentially drive individuals towards a stronger pursuit of self-fulfilment. Furthermore, lower self-confidence might be linked to greater resistance to novelty (Cho et al., 2021; Murtagh et al., 2012), since individuals with lower self-efficacy might perceive changes as stressful and overwhelming. Moreover, Self-Efficacy levels correlated positively with entrepreneurial orientation. Indeed, high self-efficacy motivates individuals to embrace new challenges and seek opportunities for growth and innovation, both of which are useful characteristics for successful entrepreneurs (Gielnik et al., 2020; Newman et al., 2019).

As it pertains to Insight Orientation, this scale showed a positive association with both Part A (Evolutionary Thrust) and Part B (Propensity for Transformation), with the exception of Distress to Change. Indeed, previous research highlighted that insight may be significantly related to predisposition to and acceptance to change (Gori et al., 2022a; Gori & Topino, 2020). Insight Orientation also showed significant and positive correlations with two work attitudes in Part C, namely Social Service and Care and Entrepreneurship. These findings support the notion that a reflective tendency is associated with a greater propensity for care work settings (Gori et al., 2022b, 2023) and entrepreneurial roles (Hjelle, 2024).

Regarding Resilience, significant positive correlations were shown with both Part A (Evolutionary Thrust) and Part B (Propensity for Transformation), except for Internal Drive for Realization, Propensity for Transformation, and Distress to Change. Indeed, previous scientific research showed a bidirectional relationship between resilience and meaning or self-fulfilment (Lewis & Hill, 2021; Miao & Cao, 2024). On the other hand, perceiving a drive to achieve a sense of purpose that still appears distant can be associated with feelings of fear and distress, which are negatively correlated with resilience (Folkman & Lazarus, 1986; Tugade & Fredrickson, 2004; Southwick et al., 2014). Moreover, individuals resistant to novelty often struggle in new situations, hindering the development of skills crucial for adapting to adversity. This limited adaptability can consequently diminish their overall resilience (Oreg & Goldenberg, 2015). Finally, higher resilience scores were correlated with a greater propensity for activities related to the Social Service and Care area, Entrepreneurship, and Customer Service and Hospitality. This suggests that an orientation towards these work areas may imply a tendency to recover more easily from challenges and setbacks that can characterize these occupations (Debreceni & Fekete-Frojimovics, 2023; Hart et al., 2014; Korber & McNaughton, 2017).

### 4.3. Limitations and Suggestions for Future Research

Within this study, several limitations should be acknowledged, and potential avenues for future research in this domain should be explored. First, a snowball sampling method was implemented, and this may have introduced potential biases due to its non-random nature. A randomized sampling method could be employed in future research to address this limitation and enhance the representativeness of the sample, providing more generalizable results. Second, there was a gender imbalance among the participants, which could affect the generalizability of the findings to the entire population. Future research should aim to recruit a more balanced sample in terms of gender to ensure that the findings are applicable across different gender groups. Moreover, only self-report instruments were used to collect data. While self-report measures are valuable for capturing personal perceptions and experiences, they are also susceptible to biases such as social desirability and inaccurate self-assessment. Future studies should consider incorporating a variety of data collection methods, such as observational data, peer evaluations, and objective performance measures, to complement self-report data. Additionally, although convergent validity was tested through correlations with theoretically related constructs such as life satisfaction, career adaptability, and self-efficacy, future research could benefit from including established self-actualization measures to strengthen convergent validation more directly. Another limitation concerns the lack of test–retest reliability data. Although the internal consistency of the META was thoroughly evaluated and found to be satisfactory, the temporal stability of the measure remains to be assessed. Future studies should therefore implement a follow-up administration to calculate stability coefficients for each META dimension and further strengthen its psychometric validation. In addition, while factorial validity and reliability were assessed through Classical Test Theory methods (EFA and CFA), item-level analyses based on Item Response Theory (IRT) were not performed. Given the multidimensional structure of the META, future studies could adopt IRT and Multidimensional IRT (MIRT) models to enhance construct precision and parameter invariance. Furthermore, no Differential Item Functioning (DIF) analyses were conducted, which limits the assessment of measurement equivalence across groups. Investigating DIF (particularly in relation to gender) should be a priority in future research to ensure fairness and generalizability.

### 4.4. Practical Implications

The *Measurement for Evolution, Transformation, and Autorealization (META)* may practically encourage and support the development of practical activities across different fields. For example, by providing a comprehensive and nuanced assessment of self-realization, the META can be used by career counsellors to better understand clients’ intrinsic motivations and propensities, thereby guiding them towards career paths that align with their aspirations and strengths (Dik et al., 2009). Similarly, psychologists and psychotherapists can use the META to identify areas where individuals may need support in their progress towards self-fulfilment. This targeted approach facilitates the development of more effective interventions, empowering individuals to achieve a fulfilling life (Wong, 2015). Furthermore, organizations can incorporate the META into their employee development programs to enhance job satisfaction and performance by aligning job roles with employees’ self-realization tendencies. This may promote the composition of a more engaged, resilient, and healthy workforce, which supports the development of the whole organization (Di Fabio, 2017; Żołnierczyk-Zreda, 2020).

## 5. Conclusions

The Measurement for Evolution, Transformation, and Autorealization (META) is a new self-report instrument developed to assess an individual’s subjective propensity for self-realization. Grounded in current scientific evidence and conceptualizations of self-realization, the META comprises three core sections: Evolutionary Thrust (Part A); Transformative Adaptation (Part B), and Work Attitude (Part C). This instrument presented excellent psychometric properties, with good indications of validity and reliability, as well as relevant correlations with indicators of self-realization and well-being outcomes. As a multidimensional and empirically grounded tool, the META contributes meaningfully to the field of self-realization research. Its comprehensive structure may allow for a nuanced understanding of how individuals engage in personal growth, transformation, and purpose-driven living. Beyond its scientific value, the META may hold strong potential for use in clinical, educational, and developmental contexts, offering practitioners a reliable means to explore and support individuals’ pathways toward authentic self-fulfilment and psychological flourishing. In doing so, the META marks a valuable step forward in bridging research and practice in the study of self-realization.

## Figures and Tables

**Table 1:** Demographic characteristics of the sample (*N* = 634).

Characteristics		*M* ± *SD*	*N* (%)
Age		37.32 ± 14.965	
Sex			
	*Males*		172 (27.1%)
	*Females*		462 (72.9%)
Marital Status			
	*Single*		342 (53.9%)
	*Married*		170 (26.8%)
	*Cohabiting*		76 (12.0%)
	*Separated*		15 (2.4%)
	*Divorced*		22 (3.5%)
	*Widowed*		9 (1.4%)
Education			
	*Elementary School diploma*		2 (0.3%)
	*Middle School diploma*		45 (7.1%)
	*High School diploma*		257 (40.5%)
	*University degree*		138 (21.8%)
	*Master’s degree*		134 (21.1%)
	*Post-lauream specialization*		58 (9.1%)
Occupation			
	*Student*		150 (23.7%)
	*Working student*		65 (10.3%)
	*Artisan*		12 (1.9%)
	*Employee*		241 (38.0%)
	*Entrepreneur*		22 (3.5%)
	*Freelance*		40 (6.3%)
	*Retired*		31 (4.9%)
	*Trader*		8 (1.3%)
	*Religious*		3 (0.5%)
	*Manager*		12 (1.9%)
	*Unemployed*		50 (7.9%)

**Table 2:** Internal consistency, Inter-Factor Correlations and HTMT analysis for each section of the Measurement for Evolution, Transformation, and Autorealization (META).

Section	Factors	Items (N)	α	ω	Inter-Factor Correlations (Above the Diagonal) and HTMT Analysis (Below the Diagonal).				
					1	2	3	4	5
***Part A***	*N* = 5	15	0.806	0.828					
Evolutionary Thrust	1. Sense of life	3	0.884	0.886	—	0.455	0.491	0.540	0.033
	2. Spirit of service	3	0.861	0.862	0.370	—	0.298	0.236	0.270
	3. Self-Authorizing	3	0.845	0.846	0.483	0.189	—	0.513	0.098
	4. Self-Centering	3	0.837	0.840	0.551	0.225	0.505	—	−0.010
	5. Internal drive for realization	3	0.657	0.694	0.096	0.224	0.028	0.009	—
***Part B***	*N* = 4	12	0.721	0.754					
Transformative Adaptation	1. Propensity for transformation	3	0.840	0.847	—	0.120	0.026	0.245	—
	2. Distress to change	3	0.800	0.801	−0.158	—	−0.478	−0.314	—
	3. Adaptability	3	0.733	0.745	0.035	0.560	—	0.522	—
	4. Fullness of the experience	3	0.617	0.645	0.242	0.336	0.590	—	—
***Part C***	*N* = 5	20	0.794	0.840					
Work Attitude	1. Social Service and Care	4	0.945	0.945	—	−0.093	0.290	0.290	0.155
	2. Administrative and Office Works	4	0.932	0.934	0.079	—	0.132	0.132	0.044
	3. Entrepreneurship	4	0.922	0.925	0.042	0.062	—	0.137	0.026
	4. Customer Service and Hospitality	4	0.919	0.923	0.215	0.129	0.129	—	0.338
	5. Manual activities	4	0.887	0.888	0.136	0.028	0.028	0.287	—

**Table 3:** Fit statistics of the META parts for three different models and chi-square variation test.

	*χ* ^2^	*df*	*p*	*CMIN/DF*	*GFI*	*TLI*	*CFI*	*RMSEA*	*SRMR*	*Models Comparison*	Δ*χ*^2^	Δ*df*	*p*
**Part A: Evolutionary Thrust**													
*Correlational Model*	169.923	80	<0.001	2.124	0.935	0.945	0.958	0.060	0.065				
*Unifactorial Model*	1203.011	90	<0.001	13.367	0.636	0.391	0.478	0.198	0.149				
										M1-M2	1033.088	10	<0.001
**Part B: Transformative Adaptation**													
*Correlational Model*	165.171	48	<0.001	3.441	0.927	0.863	0.900	0.088	0.069				
*Unifactorial Model*	694.197	54	<0.001	12.855	0.715	0.333	0.455	0.194	0.156				
										M1-M2	529.026	6	<0.001
**Part C: Work Attitude**													
*Correlational Model*	561.338	160	<0.001	3.508	0.841	0.912	0.926	0.089	0.044				
*Unifactorial Model*	4392.872	170	<0.001	25.84	0.417	0.129	0.221	0.280	0.253				
										M1-M2	4227.701	10	<0.001

*χ*^2^ = Chi-square value of model fit; *df* = degree of freedom; GFI = Goodness of Fit; TLI = Tucker Lewis index; CFI = Comparative Fit Index, RMSEA = the Root Mean Square Error of Approximation; SRMR = Standardized Root Mean Square Residual; Δ*χ*^2^ = Difference in *χ*^2^ values between the compared models; Δ*df* = Difference in number of degrees of freedom between the compared models; M1 = Correlational Model; M2 = Unifactorial Model.

**Table 4:** Pearson’s correlations and Heterotrait–Monotrait (HTMT) correlation ratio (in round brackets).

	Part A: Evolutionary Thrust						Part B: Transformative Adaptation					Part C: Work Attitude				
	Total	A1	A2	A3	A4	A5	Total	B1	B2	B3	B4	C1	C2	C3	C4	C5
Satisfaction with life (SWLS)	0.526 ** (0.609)	0.612 ** (0.695)	0.189 ** (0.219)	0.452 ** (0.520)	0.490 ** (0.574)	−0.275 ** (0.313)	0.001 (0.071)	−0.489 ** (0.566)	−0.186 ** (0.222)	0.279 ** (0.339)	0.224 ** (0.291)	−0.017 (0.018)	0.060 (0.067)	0.068 (0.079)	−0.031 (0.035)	0.034 (0.041)
Career Adaptability (CAAS)	0.613 ** (0.697)	0.418 ** (0.456)	0.287 ** (0.322)	0.502 ** (0.559)	0.444 ** (0.502)	0.165 ** (0.260)	0.353 ** (0.481)	−0.016 (0.017)	−0.135 ** (0.158)	0.474 ** (0.575)	0.429 ** (0.568)	0.035 (0.040)	0.090 * (0.097)	0.193 ** (0.207)	0.036 (0.039)	0.037 (0.043)
Concern (CAAS)	0.507 ** (0.587)	0.314 ** (0.354)	0.187 ** (0.213)	0.399 ** (0.457)	0.362 ** (0.422)	0.244 ** (0.354)	0.230 ** (0.470)	0.067 (0.076)	0.012 (0.014)	0.308 ** (0.419)	0.304 ** (0.381)	0.002 (0.003)	0.087 * (0.095)	0.231 ** (0.255)	−0.022 (0.026)	−0.046 (0.050)
Control (CAAS)	0.504 ** (0.615)	0.397 ** (0.457)	0.235 ** (0.282)	0.464 ** (0.556)	0.386 ** (0.464)	−0.010 (0.045)	0.274 ** (0.276)	−0.123 ** (0.138)	−0.198 ** (0.240)	0.419 ** (0.437)	0.316 ** (0.540)	−0.001 (0.000)	0.050 (0.057)	0.096 * (0.116)	0.085 * (0.098)	0.086 * (0.102)
Curiosity (CAAS)	0.532 ** (0.639)	0.327 ** (0.372)	0.289 ** (0.335)	0.404 ** (0.470)	0.374 ** (0.443)	0.203 ** (0.323)	0.388 ** (0.553)	0.055 (0.066)	−0.138 ** (0.166)	0.438 ** (0.635)	0.463 ** (0.551)	0.093 * (0.103)	0.039 (0.045)	0.190 ** (0.216)	0.040 (0.043)	0.051 (0.059)
Confidence (CAAS)	0.508 ** (0.595)	0.368 ** (0.413)	0.260 ** (0.295)	0.416 ** (0.476)	0.365 ** (0.426)	0.096 * (0.164)	0.301 ** (0.381)	−0.067 (0.076)	−0.150 ** (0.177)	0.438 ** (0.491)	0.361 ** (0.543)	0.030 (0.032)	0.123 ** (0.134)	0.114 ** (0.130)	0.028 (0.030)	0.046 (0.051)
General Self-Efficacy (GSES)	0.445 ** (0.514)	0.427 ** (0.479)	0.124 ** (0.144)	0.403 ** (0.468)	0.388 ** (0.455)	−0.080 * (0.061)	0.380 ** (0.505)	−0.131 ** (0.148)	−0.363 ** (0.429)	0.505 ** (0.624)	0.317 ** (0.417)	−0.003 (0.005)	0.059 (0.067)	0.247 ** (0.278)	0.033 (0.035)	0.066 (0.073)
Insight Orientation (IOS)	0.531 ** (0.673)	0.438 ** (0.533)	0.277 ** (0.339)	0.445 ** (0.551)	0.362 ** (0.457)	0.043 (0.107)	0.341 ** (0.496)	−0.038 (0.053)	−0.186 ** (0.240)	0.454 ** (0.600)	0.371 ** (0.529)	0.085 * (0.100)	0.055 (0.063)	0.222 ** (0.266)	0.073 (0.082)	0.062 (0.074)
Resilience (CD-RISC-10)	0.483 ** (0.588)	0.473 ** (0.544)	0.246 ** (0.296)	0.382 ** (0.456)	0.377 ** (0.454)	−0.082 * (0.050)	0.442 ** (0.611)	−0.142 ** (0.157)	−0.435 ** (0.530)	0.555 ** (0.707)	0.374 ** (0.515)	0.103 ** (0.119)	0.049 (0.051)	0.212 ** (0.246)	0.100 * (0.110)	0.076 (0.086)

**. Correlation is significant at the 0.01 level (2-tailed). *. Correlation is significant at the 0.05 level (2-tailed). Bold values indicate significant correlations. A1 = Sense of Life; A2 = Spirit of Service; A3 = Self-Authorizing; A4 = Self-Centering; A5 = Internal Drive for Realization; B1 = Propensity for Transformation; B2 = Distress to Change; B3 = Adaptability; B4 = Fullness of the Experience; C1 = Social Service and Care; C2 = Administrative and Office Works; C3 = Entrepreneurship; C4 = Customer Service and Hospitality; C5 = Manual activities.

## Data Availability

The data presented in this study is available on request from the corresponding author. The data is not publicly available due to privacy reasons.

## Supplementary Materials

The following supporting information can be downloaded at https://www.mdpi.com/article/10.3390/bs15070942/s1, Table S1. Descriptive and Item-Level Statistics for Part A—Evolutionary Thrust; Table S2. Descriptive and Item-Level Statistics for Part B—Transformative Adaptation; Table S3. Descriptive and Item-Level Statistics for Part C—Work Attitude.
