# Supplementary material for: META—Measurement for Evolution, Transformation, and Autorealization: A New Assessment Protocol
Source: Behav Sci (Basel). 2025 Jul 11;15(7):942. doi: 10.3390/bs15070942 (PMC12293060; doi:10.3390/bs15070942)
Supplement: Supplementary file 1 [file behavsci-15-00942-s001.zip › behavsci-3675419-supplementary.pdf]

Supplementary Table S1. Descriptive and Item-Level Statistics for Part A – Evolutionary Thrust

Factor	Item	M	SD	% Resp. 1	% Resp. 2	% Resp. 3	% Resp. 4	% Resp. 5	Item Total-Correlations
1. Sense of life	Item 1	3.28	1.08	5.2 %	18.8 %	33.1 %	28.7 %	14.2 %	0.743
	Item 6	3.23	1.09	6.9 %	17.7 %	32.3 %	31.4 %	11.7 %	0.819
	Item 11	3.40	1.07	4.7 %	15.1 %	31.2 %	33.4 %	15.5 %	0.764
2. Spirit of service	Item 2	4.30	0.728	0.3 %	1.3 %	10.3 %	44.3 %	43.8 %	0.726
	Item 7	4.16	0.805	0.5 %	2.2 %	16.1 %	43.1 %	38.2 %	0.764
	Item 12	4.28	0.778	0.3 %	2.1 %	12.0 %	40.2 %	45.4 %	0.722
3. Self-Authorizing	Item 3	4.11	0.916	0.3 %	1.1 %	3.3 %	11.8 %	83.4 %	0.716
	Item 8	3.83	1.021	1.7 %	9.3 %	23.8 %	34.7 %	30.4 %	0.713
	Item 13	4.01	0.958	0.8 %	7.3 %	18.8 %	36.4 %	36.8 %	0.707
4. Self-Centering	Item 4	3.92	0.989	1.9 %	7.3 %	20.2 %	38.6 %	32.0 %	0.682
	Item 9	3.47	1.176	7.9 %	11.7 %	27.1 %	32.0 %	21.3 %	0.716
	Item 14	3.62	1.079	4.4 %	10.1 %	27.6 %	34.9 %	23.0 %	0.712
5. Internal drive for realization	Item 5	4.01	1.100	4.3 %	5.2 %	18.6 %	28.9 %	43.1 %	0.615
	Item 10	3.54	1.204	7.4 %	12.9 %	22.4 %	32.5 %	24.8 %	0.547
	Item 15	4.60	0.681	0.5 %	0.9 %	5.5 %	23.8 %	69.2 %	0.316

Notes: All items are rated on a 5-point Likert scale ranging from 1 (“not at all”) to 5 (“very much”); Percentages indicate the proportion of participants selecting each response option. Item-total correlations are calculated with respect to each specific subdimension.

Supplementary Table S2. Descriptive and Item-Level Statistics for Part B – Transformative Adaptation

Factor	Item	M	SD	% Resp. 1	% Resp. 2	% Resp. 3	% Resp. 4	% Resp. 5	Item Total-Correlations
1. Propensity for transformation	Item 1	3.44	1.23	8.5 %	15.0 %	24.0 %	29.5 %	23.0 %	0.753
	Item 5	2.91	1.30	18.0 %	22.2 %	24.0 %	22.4 %	13.4 %	0.632
	Item 9	3.39	1.17	6.8 %	17.0 %	25.6 %	31.5 %	19.1 %	0.733
2. Distress to change	Item 2	3.08	1.16	8.2 %	24.1 %	33.3 %	20.0 %	14.4 %	0.626
	Item 6	2.61	1.18	19.1 %	31.2 %	27.0 %	14.8 %	7.9 %	0.651
	Item 10	2.54	1.11	18.3 %	34.5 %	27.3 %	14.4 %	5.5 %	0.658
3. Adaptability	Item 3	3.72	0.907	0.6 %	7.4 %	33.3 %	37.1 %	21.6 %	0.504
	Item 7	3.55	0.989	2.2 %	11.5 %	33.8 %	34.2 %	18.3 %	0.527
	Item 11	3.62	0.959	1.3 %	10.7 %	32.5 %	36.0 %	19.6 %	0.643
4. Fullness of the experience	Item 4	4.51	0.684	0.0 %	0.8 %	8.5 %	29.2 %	61.5 %	0.388
	Item 6	3.74	0.930	1.4 %	7.3 %	29.7 %	39.7 %	21.9 %	0.410
	Item 12	4.43	0.708	0.2 %	0.8 %	9.5 %	35.2 %	54.4 %	0.512

Notes: All items are rated on a 5-point Likert scale ranging from 1 (“not at all”) to 5 (“very much”); Percentages indicate the proportion of participants selecting each response option. Item-total correlations are calculated with respect to each specific subdimension.

Supplementary Table S3. Descriptive and Item-Level Statistics for Part C – Work Attitude

Factor	Item	M	SD	% Resp. 1	% Resp. 2	% Resp. 3	% Resp. 4	% Resp. 5	Item Total-Correlations
1. Sense of life	Item 1	2.97	1.36	18.6 %	20.7 %	22.4 %	21.3 %	17.0 %	0.886
	Item 2	3.07	1.28	13.9 %	20.8 %	26.0 %	23.3 %	15.9 %	0.833
	Item 3	2.42	1.28	29.3 %	30.4 %	18.0 %	13.4 %	8.8 %	0.914
	Item 4	2.38	1.29	32.6 %	26.0 %	20.7 %	11.8 %	8.8 %	0.836
2. Spirit of service	Item 5	3.57	1.21	7.4 %	12.5 %	22.9 %	30.6 %	26.7 %	0.886
	Item 6	3.23	1.17	9.5 %	16.4 %	30.8 %	28.7 %	14.7 %	0.731
	Item 7	3.47	1.31	10.7 %	13.2 %	22.1 %	25.9 %	28.1 %	0.898
	Item 8	3.32	1.35	12.3 %	17.8 %	21.0 %	23.2 %	25.7 %	0.850
3. Self-Authorizing	Item 9	2.68	1.33	26.2 %	20.3 %	22.7 %	20.8 %	9.9 %	0.860
	Item 10	3.13	1.34	17.7 %	12.9 %	25.1 %	27.0 %	17.4 %	0.722
	Item 11	2.58	1.36	30.3 %	20.7 %	21.6 %	16.1 %	11.4 %	0.855
	Item 12	2.59	1.38	31.2 %	19.2 %	21.0 %	16.9 %	11.7 %	0.825
4. Self-Centering	Item 13	2.44	1.25	31.2 %	21.9 %	24.8 %	15.9 %	6.2 %	0.878
	Item 14	2.77	1.30	22.9 %	19.6 %	25.7 %	21.8 %	10.1 %	0.716
	Item 15	2.25	1.23	36.1 %	26.8 %	18.6 %	12.8 %	5.7 %	0.880
	Item 16	2.11	1.18	39.9 %	28.2 %	17.7 %	9.1 %	5.0 %	0.793
5. Internal drive for realization	Item 17	3.09	1.37	18.1 %	17.0 %	21.3 %	24.9 %	18.6 %	0.780
	Item 18	3.09	1.34	17.2 %	17.2 %	22.1 %	26.7 %	16.9 %	0.715
	Item 19	2.86	1.38	22.2 %	19.4 %	24.9 %	17.0 %	16.4 %	0.786
	Item 20	2.69	1.36	25.7 %	22.1 %	22.9 %	15.8 %	13.6 %	0.730

Notes: All items are rated on a 5-point Likert scale ranging from 1 (“not at all”) to 5 (“very much”); Percentages indicate the proportion of participants selecting each response option. Item-total correlations are calculated with respect to each specific subdimension.
